# Dismissing Appropriate Statistical Analysis

**DOI:** 10.5812/hepatmon.7500

**Published:** 2012-11-30

**Authors:** Mohammad Reza Ghadir

**Affiliations:** 1 Gastroenterology Section, Department of Internal Medicine, Faculty of Medicine, Qom University of Medical Sciences, Qom, IR Iran

**Keywords:** Hepatitis D, Hepatitis B Virus, Prevalence, Iran

Dear Editor,

We are thankful to the referees Dr. Mohamad Amin Pourhoseingholi et al. and the Editor for pointing out some valuable suggestion and important notes needed in the manuscript (1). The major concern of reviewer was about the positive Hepatitis D patient (2). Due to high number of cases in our study, the prevalence of Hepatitis D is very low (3).

1) The only positive Hepatitis D is a 31 year-old woman. We have mentioned that she was an Afghanian lady but we have considered her native because she has Iranian ID Card, although we have not assessed her ancestry.

2) As the reviewer has mentioned, chi square test is impractical in the present study so fisher exact test is used. We have mentioned to this important note in the methods and conclusion sections.

3) It has mentioned that repeated test is performed to assess association between Hepatitis D and Tatto. So P value of likelihood ratio is not reported and both of P values are related to fisher exact test (3).
